# Lenvatinib versus bevacizumab when combined with PD-1/L1 inhibitor and hepatic arterial infusion chemotherapy in unresectable hepatocellular carcinoma

**DOI:** 10.3389/fimmu.2025.1573098

**Published:** 2025-05-23

**Authors:** Lichang Huang, Yujie Xu, Na Liu, Hailong Chen, Zichao Wu, Qijiong Li, Minqiang Lu, Wei Wei, Yaojun Zhang, Minshan Chen, Li Xu, Ming Shi, Zhicheng Lai

**Affiliations:** ^1^ Department of Hepatobiliary Oncology, State Key Laboratory of Oncology in South China, Guangdong Provincial Clinical Research Center for Cancer, Sun Yat-sen University Cancer Center, Guangzhou, China; ^2^ Department of Hepatobiliary Pancreatic Surgery, Guangzhou First People’s Hospital, Guangzhou, Guangdong, China; ^3^ Department of Hepatobiliary Surgery, The Second Affiliated Hospital of Guangzhou Medical University, Guangzhou, China

**Keywords:** hepatocellular carcinoma, lenvatinib, bevacizumab, combination therapy, potential resectable

## Abstract

**Introduction:**

The combination of anti-angiogenic agents, PD-1/L1 inhibitors, and hepatic arterial infusion chemotherapy (HAIC) has emerged as an important strategy for unresectable hepatocellular carcinoma (uHCC), yet comparative data on efficacy and safety between different anti-angiogenic agents (lenvatinib [LenHAP] or bevacizumab [BevHAP]) remain lacking, especially in patients with potential resectable features (PotenR).

**Methods:**

This retrospective study included patients from 3 hospitals. Included patients received LenHAP or BevHAP as the first-line treatment. The overall survival (OS), progression-free survival (PFS), objective response rate (ORR), conversion resection rate (CRR) and adverse events (AE) were compared.

**Results:**

We included 108 uHCC patients in each group after propensity score matching (PSM), of which PotenR patients accounted for 34.3%. Compared with BevHAP group, the LenHAP group demonstrated significantly prolonged median PFS (12.6 *vs.* 8.1 months; HR, 0.64; 95% CI, 0.46-0.90; *p*=0.0085), with a trend toward improved OS (26.4 *vs.* 19.6 months; HR, 0.71; 95% CI, 0.41-1.1; *p*=0.091). PotenR patients receiving LenHAP achieved superior outcomes, including markedly extended OS (both not reached in median, *p*=0.018), PFS (19.8 *vs.* 11.5, months, *p*=0.0067), and higher conversion resection rates (52.6% *vs.* 25.0%, *p*=0.015). Both regimens showed comparable safety profiles, with similar frequencies of grade 3–4 adverse events (47.2% *vs.* 39.8%, *p*=0.27) and serious adverse events (4.6% *vs.* 8.3%, *p*=0.27).

**Conclusions:**

LenHAP might offer enhanced clinical benefits over BevHAP in uHCC, particularly for PotenR patients, while maintaining equivalent tolerability.

## Introduction

Hepatocellular carcinoma (HCC) has been a leading cause of cancer-related death, and most patients present with unresectable disease (1, 2). Programmed death 1/programmed death 1 ligand 1 antibody (PD-1/L1 antibody) combined with anti-angiogenic agents such as atezolizumab plus bevacizumab were recommended as the preferred first-line treatment for unresectable HCC (3–6). However, atezolizumab plus bevacizumab showed limited efficacy in high-risk unresectable HCC (7). Therefore, the combination of locoregional therapies such as hepatic arterial infusion chemotherapy (HAIC) with systemic therapies was suggested (8–10).

Several phase II studies had suggested that HAIC combined with PD-1/L1 antibodies and anti-angiogenic agents had a promising anti-tumor activity and manageable safety (11, 12). The one of the reasons for the improvement in the prognosis was that the combination therapy had a high conversion resection rate, which was reported as 17.1%-60.0% in previous studies (11–13). Therefore, identifying patients with potential resectable features (PotenR) and increasing the conversion resection rate help to further improve the prognosis of unresectable HCC patients (14, 15).

Although the triple combination regimen had been widely promoted in China (16, 17), as there were different types of PD-1/L1 antibodies and anti-angiogenic agents, this regimen had various drug combination options. Recently, two studies had compared the efficacy and safety of PD-1 antibodies and PD-L1 antibodies in the combination therapy (18, 19). However, objective data is still missing to compare the differences between lenvatinib (LenHAP) or bevacizumab (BevHAP) in the combination therapy for unresectable HCC, especially in PotenR patients. Since the mechanisms are different between lenvatinib and bevacizumab, the efficacy as part of a combination therapy may also vary.

Therefore, we conducted this first study to compare the efficacy and safety of lenvatinib with bevacizumab when combined with PD-1/L1 antibodies and HAIC in unresectable HCC.

## Materials and methods

### Patients

This retrospective, multi-center and propensity score matching (PSM) study was conducted following the International Conference on Harmonisation Guidelines for Good Clinical Practice and the principles of the Declaration of Helsinki at three medical sites. This study was approved by institutional review board and the ethics committee (B2022-301-01). HAIC was recommended as the locoregional therapy according to our previous studies (20, 21). Lenvatinib combined with PD-1/L1 antibodies (LenHAP) were recommended as the systemic therapies based on previous studies (11, 22). Bevacizumab (BevHAP) combined with atezolizumab or sintilimab were recommended based on IMbrave 150 study and ORIENT-32 study (3, 6). All patients had the final decision, and gave written informed consent.

The main inclusion criteria were as follows: patients aged 18 years or older, with unresectable, locally advanced, or metastatic HCC, with the diagnosis confirmed by histologic or cytologic analysis or clinical features according to the American Association for the Study of Liver Disease criteria (23), who had received no previous treatment, had at least on measurable disease, as defined by Response Evaluation Criteria In Solid Tumours version 1.1 (RECIST v1.1) criteria (24), had a baseline Eastern Cooperative Oncology Group (ECOG) performance status of 0 or 1, had a Child-Pugh liver function score of 7 or less and had adequate hematologic and organ function (absolute neutrophil count ≥1.2×10^9^/l, platelet count ≥60×10^9^/l, total bilirubin < 30μmol/l, albumin ≥ 30g/l, aspartate transaminase and alanine transaminase ≤ 5×upper limit of the normal, creatinine clearance rate of ≤ 1.5×upper limit of the normal, and left ventricular ejection ≥ 45%). The key exclusion criteria were history of HIV, organ allograft, combined with other malignant tumors, evidence of hepatic decompensation, bleeding diathesis or event, and allergy to the investigational agents or any agent given in association with this trial and incomplete medical information.

Potentially resectable features (PotenR) were defined as follows according to the Chinese expert consensus on Neoadjuvant and Transforming Therapy for Hepatocellular carcinoma (version 2023): the tumors were localized in the same segment or half of liver (or the lesions outside the resection area could be treated by ablation at the same time) and were consistent with resectable hepatic vein invasion, portal vein invasion (PVTT) (except main PVTT) or bile duct tumor thrombus without distant metastasis (25).

### Treatments

In the LenHAP group, patients initiated lenvatinib (12 mg/day [for bodyweight ≥60kg] or 8 mg/day [for bodyweight <60kg]) 3–7 days before initial HAIC to confirm tolerability and then underwent 21-day treatment cycles of lenvatinib, PD-1/L1 antibody, and HAIC (11, 21, 22). In the BevHAP group, patients received 15 mg/kg body weight of bevacizumab and PD-1/L1 antibody intravenously followed by HAIC every 21 days (**Supplementary Figure S1**). HAIC was performed as follows. A catheter will be superselectively placed into the feeding arteries of the tumor and the tumor thrombus. And the patients were transferred to inpatient ward for drug infusion (FOLFOX regimen, oxaliplatin, leucovorin and 5-fluorouracil) via the hepatic artery. After HAIC was completed, the catheter and sheath were removed (20, 26). The detailed procedures, dose reduction, interruption, discontinuation of the therapy and post-study treatment were described in eMethods.

### Outcomes

The primary outcome was overall survival (OS), and the secondary outcomes were progression-free survival (PFS), objective response rate (ORR), disease control rate (DCR), duration of response (DoR), conversion resection rate, and adverse events. The definition of outcomes was described in detail in eMethods.

### Statistical analyses

PSM analysis was conducted to reduce the influence of selection bias. The following parameters were included in the PSM: ECOG PS, Child-Pugh grade, absence or presence of PVTT, absence or presence of hepatic vein/inferior vena cava tumor thrombus (HVTT), absence or presence of metastasis, tumor size, tumor number, and serum AFP level. Matched pairs were then formed using a 1-to-1 nearest-neighbor caliper width of 0.1.

All included patients received at least one cycle of LenHAP or BevHAP, and the analysis was performed on a per-protocol basis. We used SPSS (version 25.0) and R studio (R version 4.3.0) for all analyses. The results are reported as the mean (standard deviation [SD]), number (%), or median (95% confidence interval [CI]) and were compared by Student’s t-tests, Mann-Whitney U test, or chi-square tests. The PFS and OS with associated 95% CIs were analyzed by the Kaplan–Meier method and were compared between treatment groups with the use of a log-rank test. Subgroup analyses were performed across different clinical characteristics, and hazard ratios (HR) for disease progression or death were estimated with a Cox proportional hazards model. All *p* values were two-sided, with *p* values less than 0.05 considered significant.

## Results

**Figure 1:**
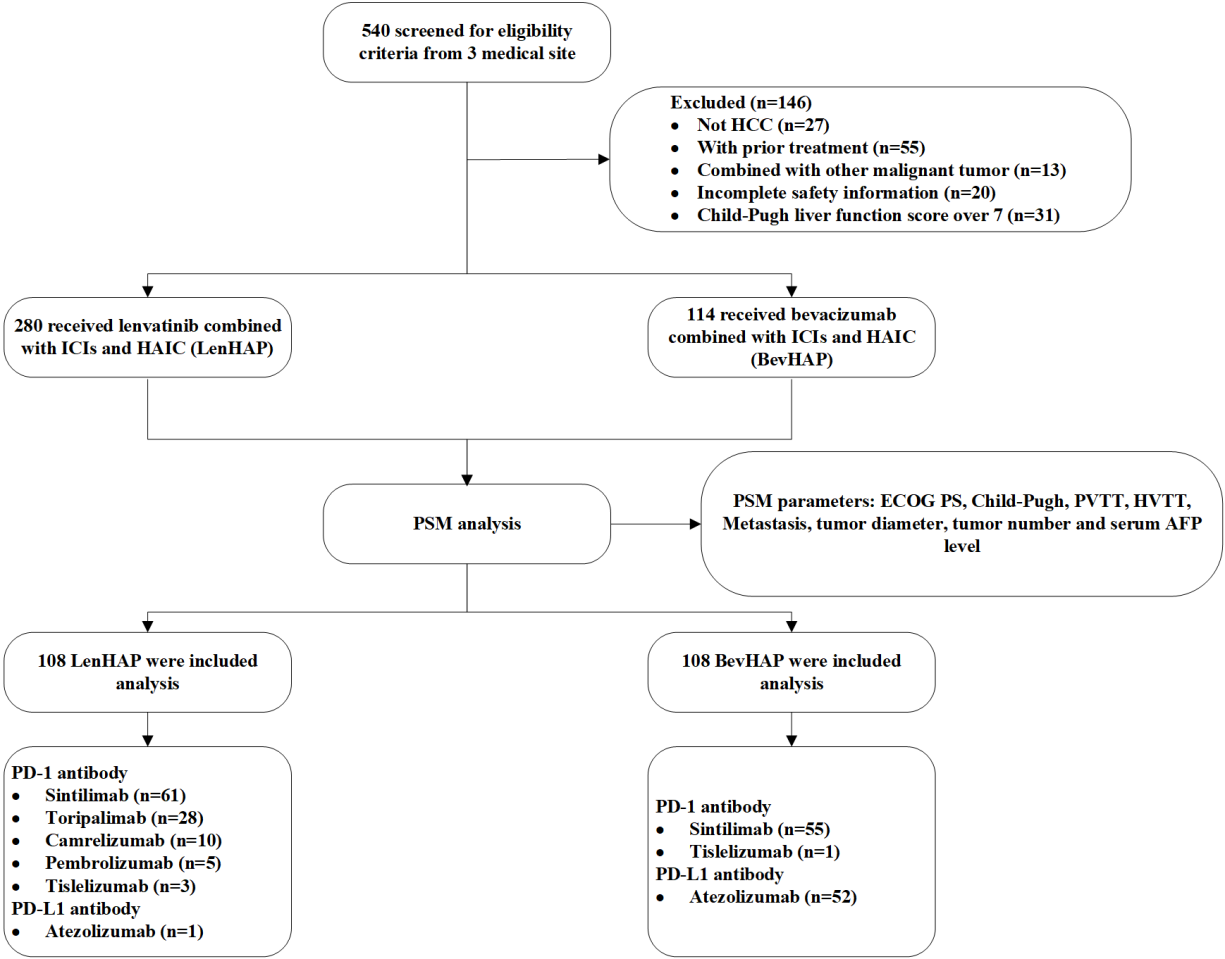
Flow chart. AFP, alpha-fetoprotein; HAIC, hepatic arterial infusion chemotherapy; HCC, hepatocellular carcinoma; HVTT, hepatic vein/inferior vena cava tumor thrombus; ICI, Immune checkpoint inhibitors; PD-1, programmed cell death protein 1; PD-L1, programmed cell death 1 ligand 1; PSM, propensity score matching; PVTT, portal vein invasion.

Between March 29, 2019, and September 25, 2023, a total of 540 patients were screened for inclusion in this study. After PSM analysis, we finally included 216 unresectable HCC patients (**Figure 1**). The follow-up went to August 7, 2024. There were 75.5% of patients with BCLC C stage, including 46.8% of patients with Vp3–4 and 31.5% with metastasis. About 34.3% of patients were considered with potentially resectable features. 99.1% of patients received PD-1 antibodies in the LenHAP group, compared with 51.9% of patients in the BevHAP group (*p*<0.001). There was no significant difference in baseline characteristics between the two groups, such as age (*p*=0.20), Child-Pugh grade (*p*=0.28), tumor size (*p*=0.13), PVTT grading (*p*=0.89), extrahepatic metastasis (*p*=0.56) and potential resectable group (*p*=0.77). And the use of PD-1/L1 antibodies were also listed in **Table 1**.

**Table 1:** Baseline characteristics.

Characteristics	LenHAP (n=108)	BevHAP (n=108)	*p*
Age			0.20
≤50	44 (40.7%)	35 (32.4%)	
>50	64 (59.3%)	73 (67.6%)	
Sex			0.25
male	100 (92.6%)	95 (88.0%)	
female	8 (7.4%)	13 (12.0%)	
HBsAg			0.14
Positive	92 (85.2%)	99 (91.7%)	
Negative	16 (14.8%)	9 (8.3%)	
ECOG PS			1.0
0	104 (96.3%)	104 (96.3%)	
1	4 (3.7%)	4 (3.7%)	
Child-Pugh grade			0.28
A	106 (98.1%)	102 (94.4%)	
B	2 (1.9%)	6 (5.6%)	
ALBI grade			0.68
1	67 (62.0%)	64 (59.3%)	
2	40 (37.0%)	44 (40.7%)	
3	1 (0.9%)	0 (0%)	
ALB, median (IQR), g/dL	41.7 (38.4-43.7)	41.9 (37.9-44.9)	0.66
ALT, median (IQR), U/L	45.0 (30.4-63.8)	45.7 (29.9-64.2)	0.78
AST, median (IQR), U/L	65.1 (40.9-105.0)	63.7 (38.1-104.9)	0.85
TBIL, median (IQR), µmol/L	16.1 (12.5-22.0)	15.7 (11.8-24.5)	0.91
PT, median (IQR), s	12.2 (11.7-12.9)	12.2 (11.3-12.7)	0.26
Tumor size, cm, mean (SD)	11.0 (3.9)	10.2 (5.0)	0.094
≤10	50 (46.3%)	61 (56.5%)	0.13
>10	58 (53.7%)	47 (43.5%)	
Tumor number			0.48
≤3	35 (32.4%)	40 (37.0%)	
>3	73 (67.6%)	68 (63.0%)	
PVTT (Japan)			
Vp0	43 (39.8%)	45 (41.7%)	0.89
Vp1-2	15 (13.9%)	12 (11.1%)	
Vp3	21 (19.4%)	24 (22.2%)	
Vp4	29 (26.9%)	27 (25.0%)	
HVTT			0.33
No	93 (86.1%)	93 (86.1%)	
Hepatic vein	7 (6.5%)	11 (10.2%)	
Inferior vena cava	8 (7.4%)	4 (3.7%)	
Extrahepatic metastasis			0.56
No	76 (70.4%)	72 (66.7%)	
Yes	32 (29.6%)	36 (33.3%)	
AFP, ng/ml, median (IQR)	1097 (40.3-25969)	550 (21.1-11523)	0.36
≤400	48 (44.4%)	52 (48.1%)	0.59
>400	60 (55.6%)	56 (51.9%)	
BCLC stage			0.43
A or B	29 (26.9%)	24 (22.2%)	
C	79 (73.1%)	84 (77.8%)	
Potentially resectable*			0.77
No	70 (64.8%)	72 (66.7%)	
Yes	38 (35.2%)	36 (33.3%)	
PD-1/L1 antibody			*p*<0.001
PD-1 antibody			
Tislelizumab	3 (2.8%)	1 (0.9%)	
Camrelizumab	10 (9.3%)	0	
Pembrolizumab	5 (4.6%)	0	
Toripalimab	28 (25.9%)	0	
Sintilimab	61 (56.5%)	55 (50.9%)	
PD-L1 antibody			
Atezolizumab	1 (0.9%)	52 (48.1%)	

*Potentially resectable were defined as that the tumors were localized in the same segment or half of liver (or the lesions outside the resection area could be treated by ablation at the same time) and were consistent with resectable hepatic vein invasion, portal vein invasion (PVTT) (except main PVTT) or bile duct tumor thrombus without distant metastasis.

Treatment administration was listed in **Supplementary Table S1**. The median treatment cycles were 3 in both two groups (*p*=0.55). More patients in the BevHAP group subsequently received transcatheter arterial chemoembolization (TACE) (*p*=0.037) and lenvatinib (*p*=0.035), while more patients in the LenHAP group received rivoceranib (p=0.065) and camrelizumab (p=0.007) as the second-line therapy. Importantly, subsequent radical resection was conducted for 21 patients in the LenHAP group and 11 patients in the BevHAP group (*p*=0.033).

### Efficacy

**Figure 2:**
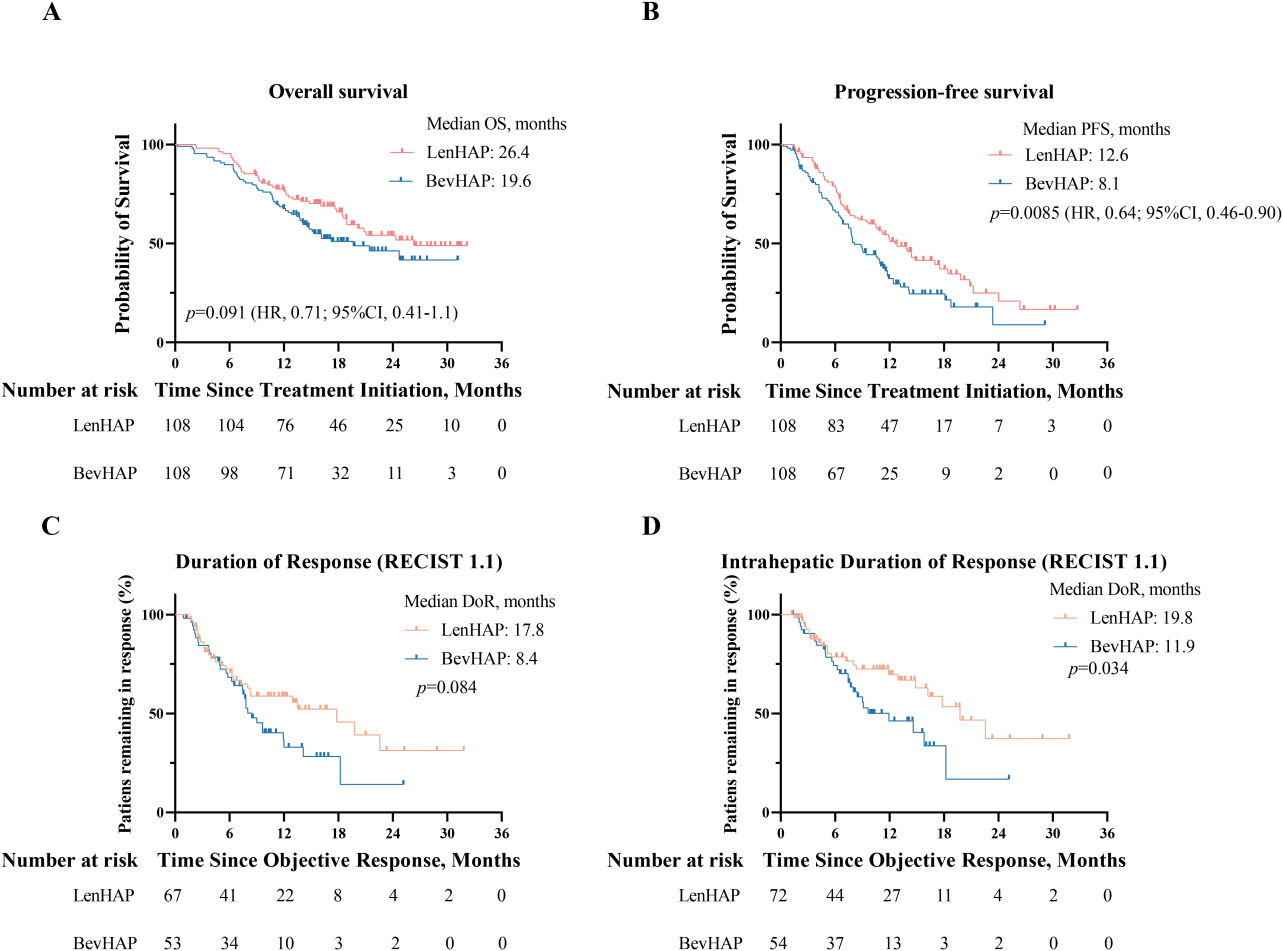
Survival analysis and duration of tumor response. **(A)** Kaplan-Meier curves of overall survival and progression-free survival **(B)**. **(C)** Duration of overall tumor response per RECIST v1.1. **(D)**. Duration of intrahepatic tumor response per RECIST v1.1.

The median OS was 26.4 months in the LenHAP group and 19.6 months (95%CI, 12.3-27.0) in the BevHAP group (HR, 0.71; 95% CI, 0.41-1.1, *p*=0.091) (**Figure 2A**). The median PFS was significantly longer with LenHAP group (12.6 months; 95% CI, 10.0-15.2) than with BevHAP group (8.1 months; 95% CI, 6.7-9.5) (HR, 0.64; 95% CI, 0.46-0.90; *p*=0.0085) (**Figure 2B**). The results of univariate and multivariate analyses of OS and PFS are listed in **Supplementary Table S2**. The presence of more than three tumors, presence of metastasis and non-PotenR were the independent risk factors for OS, while the treatment group, age less than 50, presence of more than three tumors, presence of metastasis and serum AFP level over 400 ng/ml were the independent risk factors for PFS.

The tumor response is summarized in **Table 2**. The confirmed ORR was 62.0% in the LenHAP group and 49.1% in the BevHAP group per RECIST v1.1 (*p*=0.055). And the LenHAP group achieved significantly higher confirmed ORR than BevHAP group per mRECIST (83.3% *vs.* 68.5%, *p*=0.028). Although there was no significantly difference in overall tumor DoR between the two groups, the median DoR of intrahepatic targeted lesions was significantly longer with LenHAP group than with BevHAP group (19.8 *vs.* 11.9 months, *p*=0.034) according to RECIST v1.1 (**Figures 2C, D**). Additionally, the mean reduction rate per RECIST v1.1 for intrahepatic targeted lesions was 35.7% and 30.5% of patients achieved DCR in the respective groups (*p*=0.056) (**Supplementary Figure S2A**).

**Table 2:** Tumor response.

Overall	RECIST 1.1		*p*	mRECIST		*p*
	LenHAP (n=108)	BevHAP (n=108)		LenHAP (n=108)	BevHAP (n=108)	
CR	0	0		10 (9.3%)	4 (3.7%)	0.097
PR	67 (62.0%)	53 (49.1%)	0.055	80 (74.1%)	70 (64.8%)	0.14
SD	35 (32.4%)	43 (39.8%)	0.26	12 (11.1%)	22 (20.4%)	0.062
PD	6 (5.6%)	11 (10.2%)	0.21	6 (5.6%)	11 (10.2%)	0.21
NA	0	1 (0.9%)	1	0	1 (0.9%)	1
ORR	67 (62.0%)	53 (49.1%)	0.055	90 (83.3%)	74 (68.5%)	0.011
DCR	102 (94.4%)	96 (88.9%)	0.14	102 (94.4%)	96 (88.9%)	0.14
Intrahepatic	RECIST 1.1		*p*	mRECIST		*p*
	LenHAP (n=108)	BevHAP (n=108)		LenHAP (n=108)	BevHAP (n=108)	
CR	0	0		24 (22.2%)	11 (10.2%)	0.016
PR	72 (66.7%)	54 (50%)	0.013	72 (66.7%)	63 (58.3%)	0.21
SD	34 (31.5%)	46 (42.6%)	0.091	10 (9.3%)	26 (25.9%)	0.003
PD	2 (1.9%)	7 (6.5%)	0.17	2 (1.9%)	7 (6.5%)	0.17
NA	0	1 (0.9%)	1	0	1 (0.9%)	1
ORR	72 (66.7%)	54 (50%)	0.013	96 (88.9%)	74 (68.5%)	<0.001
DCR	106 (98.1%)	100 (92.6%)	0.052	106 (98.1%)	100 (92.6%)	0.052

The OS and PFS benefited from LenHAP group compared with BevHAP group across the clinically relevant subgroups shown in **Supplementary Figures S3A, B**. The results of subgroup analyses were provided in eResults.

**Figure 3:**
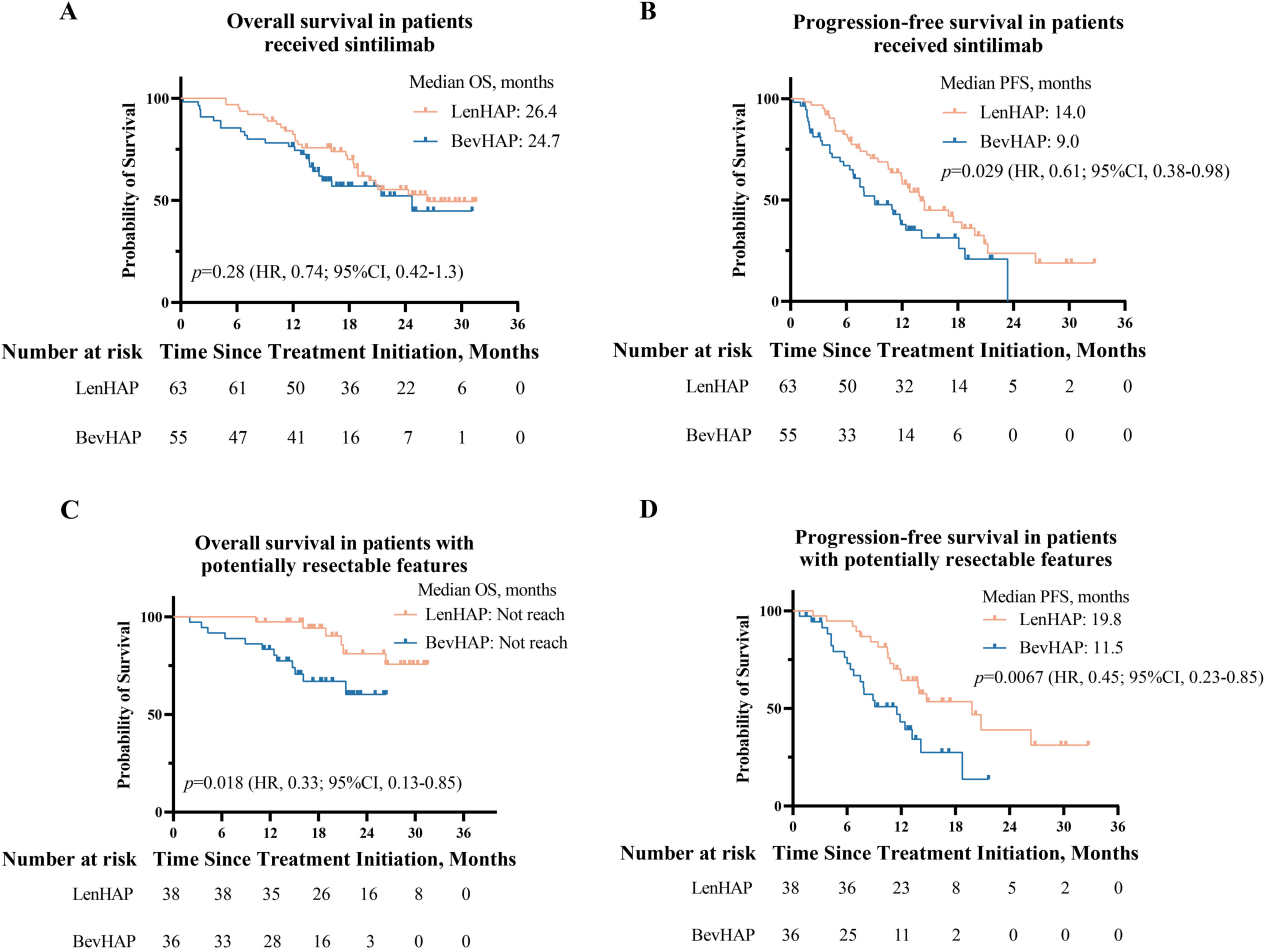
Survival analysis of patients who received sintilimab and PotenR patients. **(A)** Kaplan-Meier curves of overall survival and progression-free survival **(B)** in patients received sintilimab. **(C)** Kaplan-Meier curves of overall survival and progression-free survival **(D)** in PotenR patients. CI, confidence interval; HR. hazard ratio.

For patients treated with sintilimab, the median OS was similar between the two groups (26.4 *vs.* 24.7 months, *p*=0.28) (**Figure 3A**). And LenHAP group presented with significantly longer PFS than with BevHAP group (14.0 *vs.* 9.0 months, *p*=0.029) (**Figure 3B**). The detailed results were provided in eResults.

For PotenR patients, LenHAP therapy reduced the risk of death by 67% (HR, 0.33; 95% CI, 0.13-0.85, *p*=0.018) and the risk of disease progression by 55% (HR, 0.45; 95% CI, 0.23-0.85, *p*=0.0067) when compared with BevHAP therapy (**Figures 3C, D**). Compared with BevHAP therapy, the LenHAP therapy had a higher ORR (71.1% *vs.* 52.8%, *p*=0.11) (**Supplementary Table S5**) and had a significantly higher reduction rate of intrahepatic targeted lesions among patients achieved DCR according to RECIST v1.1 (38.7% *vs.* 30.6%, *p*=0.034, **Supplementary Figure S2C**). The conversion resection rate was significantly higher with LenHAP group than with BevHAP group (52.6% *vs.* 25.0%, *p*=0.015). In the multivariate analyses, therapy group was the independent risk factor for PFS (*p*=0.018) rather than OS (*p*=0.057) (**Supplementary Table S6**). Additionally, PotenR patients who received sintilimab also significantly benefited PFS from LenHAP (19.8 *vs.* 9.1 months, *p*=0.02), while the OS were not reached in median for both groups (**Supplementary Figures S2D, E**).

### Safety

The treatment-related AEs with high incidence rates (≥10%) are shown in **Table 3**. The frequencies of all grades (32 [32.4%] vs. 4 [3.7%]; *p*<0.001) or grade 3–4 hand-foot syndrome (8 [7.4%] vs. 0 [0%]; *p*=0.007) were significantly higher with LenHAP group than with BevHAP group. Compared with LenHAP group, patients in the BevHAP group had higher frequencies of upper gastrointestinal bleeding (0.9% *vs.* 4.6%, *p*=0.34) and anemia (65.7% *vs.* 75.9%, *p*=0.11). Three patients developed grade 1–2 immune-related adverse events (1 hepatitis and 2 dermatitis) in the LenHAP group and 3 patients (1 hepatitis, 1 hypothyroidism, and 1 nephritis) in the BevHAP group. Additionally, one patient developed a PD-L1 inhibitor allergy in the BevHAP group and subsequently stopped using it. To sum up, the total frequencies of grade 3-4 (47.2% *vs.* 39.8%, *p*=0.27) or serious adverse events (SAE) (4.6% *vs.* 8.3%, *p*=0.27) were similar between the two groups, and there was no treatment-related death in this study.

**Table 3:** Treatment-related adverse events.

Adverse Events	LenHAP (n=108)				BevHAP (n=108)				*p* for any grade	*p* for G3-G4
	any grade	grade1-2	grade3	grade4	any grade	grade1-2	grade3	grade4		
Hypertension	48 (44.4%)	32 (29.6%)	16 (14.8%)	0	46 (42.6%)	28 (25.9%)	18 (16.7%)	0	0.74	0.71
Fatigue	57 (52.8%)	50 (46.3%)	7 (6.5%)	0	54 (50.0%)	49 (45.4%)	5 (4.6%)	0	0.63	0.55
Fever	14 (13.0%)	10 (9.3%)	4 (3.7%)	0	14 (13.0%)	13 (12.1%)	1 (0.9%)	0	0.61	0.37
Sensory neuropathy	22 (20.4%)	21 (19.5%)	1 (0.9%)	0	19 (17.6%)	19 (17.6%)	0	0	0.8	1.0
Abdominal pain	35 (32.4%)	29 (26.8%)	6 (5.6%)	0	36 (33.3%)	31 (28.7%)	5 (4.6%)	0	0.62	0.76
Nausea	43 (39.8%)	39 (36.1%)	4 (3.7%)	0	38 (35.2%)	35 (32.4%)	3 (2.8%)	0	0.79	1.0
Vomit	28 (25.9%)	26 (24.1%)	2 (1.9%)	0	30 (27.8)	28 (25.9%)	2 (1.9%)	0	0.98	1.0
Diarrhea	43 (39.8%)	33 (30.6%)	10 (9.3%)	0	32 (29.6%)	27 (25.0%)	5 (4.6%)	0	0.32	0.18
Hand-foot syndrome	35 (32.4%)	27 (25.0%)	8 (7.4%)	0	4 (3.7%)	4 (3.7%)	0	0	<0.001	0.007
Neutropenia	19 (17.6%)	5 (4.6%)	9 (8.3%)	5 (4.6%)	10 (9.3%)	2 (1.9%)	3 (2.8%)	5 (4.6%)	0.26	0.18
Anemia	71 (65.7%)	71 (65.7%)	0	0	82 (75.9%)	82 (75.9%)	0	0	0.11	–
Thrombocytopenia	52 (48.1%)	44 (40.7%)	6 (5.6%)	2 (1.9%)	48 (44.4%)	41 (37.9%)	7 (6.5%)	0	0.10	1.0
Elevated ALT	72 (66.7%)	68 (63.0%)	4 (3.7%)	0	70 (64.8%)	65 (60.2%)	5 (4.6%)	0	0.38	1.0
Elevated AST	101 (93.5%)	80 (74.1%)	20 (18.5%)	1 (0.9%)	103 (95.4%)	83 (76.8%)	20 (18.5%)	0	0.84	1.0
Hyperbilirubinemia	61 (56.5%)	60 (55.5%)	0	1 (0.9%)	59 (54.6%)	57 (52.8%)	2 (1.9%)	0	0.71	1.0
Elevated CRE	7 (6.5%)	7 (6.5%)	0	0	10 (9.3%)	8 (7.4%)	2 (1.9%)	0	0.51	0.50
Urinary protein	53 (49.1%)	52 (48.1%)	1 (0.9%)	0	62 (57.4%)	57 (52.8%)	5 (4.6%)	0	0.29	0.21
Hypoalbuminemia	106 (98.1%)	106 (98.1%)	0	0	105 (97.2%)	105 (97.2%)	0	0	0.062	–
Prolonged PT	7 (6.5%)	5 (4.6%)	2 (1.9%)	0	2 (1.9%)	2 (1.9%)	0	0	0.20	0.50
Asites	7 (6.5%)	6 (5.6%)	1 (0.9%)	0	5 (4.6%)	5 (4.6%)	0	0	0.89	1.0
Upper gastrointestinal bleeding	1 (0.9%)	0	1 (0.9%)	0	5 (4.6%)	3 (2.8%)	2 (1.9%)	0	0.34	1.0
Grade 3-4	51 (47.2%)				43 (39.8%)				0.27	
Serious AE	5 (4.6%)				9 (8.3%)				0.27	

## Discussion

There is an increasing number of studies have focused on combination therapy (HAIC, anti-angiogenic agents, and PD-1/L1 antibodies) for unresectable HCC (18, 19, 27–30). Two recent retrospective studies had compared the efficacy and safety of PD-1 and PD-L1 antibodies in the combination therapy, and there was no significant difference in OS or PFS (18, 19). However, differences in the efficacy and safety of distinct anti-angiogenic agents (such as lenvatinib versus bevacizumab) in combination with PD-1/L1 antibodies and HAIC have never been determined. Our results suggest that compared with BevHAP, LenHAP significantly prolonged PFS and had a longer OS and higher ORR per RECIST v1.1. There was no significant difference in the incidence of grade 3–4 adverse events or SAE between the two groups. In subgroup analyses, patients with PotenR significantly benefited from LenHAP in terms of OS and PFS. Patients with PotenR who received LenHAP therapy had a significantly higher reduction rate of intrahepatic targeted lesions and conversion resection rates.

There was no significant difference in OS between the LenHAP group and BevHAP group, even though LenHAP tended to outperform BevHAP in the kaplan-meier curve of OS. The lack of significant difference in OS may be due to the insufficient follow-up time and subsequent treatments. On one hand, restricted by the limited period of follow-up, only 44.4% death events were observed. On the other hand, more patients in the BevHAP group received subsequent TACE and lenvatinib to control the disease progression, which contributed to noticeable survival benefit (31, 32). Our results also reflected the significantly lengthen PFS was observed in LenHAP group compared to BevHAP group. This may be due to several reasons. Firstly, LenHAP group achieved higher ORR with a longer DoR and higher reduction rate of intrahepatic targeted lesions per RECIST v1.1. Secondly, patients without HVTT or metastasis significantly benefited PFS from LenHAP, and the such patients occupied nearly 70% of the total population. Additionally, Li et al. reported that patients who had exonic nonsense or frameshift mutations of the DNA damage repair gene might contribute to response to the treatment of lenvatinib and PD-1 inhibitors (33). Oxaliplatin in HAIC contributed to damage the function of DNA mismatch repair (34), which might have a synergistic effect with lenvatinib and PD-1 inhibitors. However, it was not clear whether the damage to the function of DNA mismatch repair would affect the efficacy of bevacizumab and PD-1/L1 inhibitors.

In the multivariate analyses, the treatment group, age, tumor number, metastasis, and serum AFP level were independent risk factors for PFS, which was consistent with previous studies (29). Interestingly, patients aging over 50 had a significantly longer PFS than younger patients in this study, which might be due to a higher intrahepatic tumor burden and higher proportion of BCLC C stage in patients younger than 50. Specially, in addition to tumor numbers and metastasis, our study suggested that PotenR was also the independent risk factor for OS.

To strive opportunity for radical resection through conversion therapy can improve the prognosis of unresectable HCC. Increasing studies have focused on the conversion resection rate of anti-angiogenic agents combined with ICIs and HAIC in the treatment of unresectable HCC, which varied from 12.7% to 61.1% (30, 35–38). However, there is currently no consistent standard for defining PotenR patients. With reference to the Chinses expert consensus and our clinical experience, we modified the restriction condition of PVTT to non-main PVTT in our study. The total conversion resection rate in PotenR patients was 39.2% (52.6% in LenHAP group; 25.0% in BevHAP group). The conversion resection rate of LenHAP group in our study was higher than that Dong et al. had reported, which might be due to the small sample size of our study and the different tumor characteristics (36). Although the tumor size was significantly larger in the LenHAP group (**Supplementary Table S5**), the conversion resection rate in the LenHAP group was significantly higher than that of BevHAP group, which might be attributed to the higher rate of intrahepatic ORR and significantly higher tumor reduction rate in the LenHAP group. Although the LenHAP therapy presented with significantly longer OS and PFS in PotenR patients, therapy group was only the independent risk factor for PFS rather than OS. Therefore, prospective study with large sample size would need to verify whether PotenR patients significantly benefit from LenHAP. Additionally, considering the promising anti-tumor activity of the combination therapy, whether HCC patients with Vp3–4 could be considered as PotenR patients deserves more exploration.

The safety profile of LenHAP or BevHAP therapy was generally consistent with historical data, with no new safety signals reported (3, 6, 20, 39). Anti-angiogenic agents- related adverse events were in consistent with their different structures and characteristics. LenHAP group had a higher percentage of hand-foot syndrome and diarrhea patients, while anemia and upper gastrointestinal bleeding was more frequently observed in BevHAP. This suggested that while LenHAP therapy might offer certain survival benefits, it came at the cost of more severe side effects that affected quality of life. Dose reduction of lenvatinib combined with symptomatic drugs could relief hand-food syndrome and diarrhea, while upper gastrointestinal bleeding was treated with hemostatic drugs and/or endoscopic hemostasis. Additionally, lenvatinib-associated hepatic encephalopathy was not observed in our study. There was no grade 3–4 immune-related adverse event in this study. ICI therapy was paused in patients who developed grade 1–2 immune-related adverse events and hormone therapy was given. After the symptoms were relieved, researchers decided whether ICI therapy could continue.

This study had several limitations. First, this was a retrospective study with a limited sample size, which might affect the interpretation of the results. Therefore, we used PSM analyses to balance the baseline characteristics between the two groups and included patients from three medical sites to make the results more convincing. Second, the follow-up time was not enough to determine whether there was a statistical difference in OS between the two groups. Third, the difference in efficacy might be not only attributed to the different anti-angiogenic agents, but also the synergistic effects because of the difference in the use of ICIs between the two groups. The results of patients received sintilimab also suggested that LenHAP therapy presented with significantly longer PFS and significantly higher ORR. Fourth, our study failed to explore potential beneficiary subgroups of BevHAP regimen, and we would conduct the analyses focusing on this subgroup in the future. Fifth, because the treatment group was the independent risk factor for PFS rather than OS, prospective studies are needed to confirm whether PotenR patients benefit better prognosis from LenHAP. Finally, the study primarily included HBV-positive patients from China, which might restrict the generalizability of the conclusions to broader patient populations. Future studies should enroll diverse cohorts, including hepatitis C virus-related and non-viral etiology HCC patients, to validate whether our findings can be extrapolated to wider populations.

## Conclusion

Our results suggested that compared with BevHAP, LenHAP significantly prolonged the PFS, and had a longer OS and higher ORR per RECIST v1.1. The safety of the two groups was similar. In the subgroup analyses, PotenR patients significantly benefited OS and PFS from LenHAP, which were needed further research.

## Data Availability

The original contributions presented in the study are included in the article/**Supplementary Material**. Further inquiries can be directed to the corresponding authors.

## Supplementary material

Supplementary Figure 1 Graphical abstract of two treatment groups.

Supplementary Figure 2 Reduction rate of intrahepatic target lesions, and survival analysis in PotenR patients received sintilimab. **(A)** Reduction rate of intrahepatic target lesions per RECIST v1.1 in all patients who achieved disease control, in patients who received sintilimab and achieved disease control **(B)**, and in PotenR patients who achieved disease control **(C)**. **(D)** Kaplan-Meier curves of overall survival and progression-free survival **(E)** in PotenR patients who received sintilimab.

Supplementary Figure 3 Subgroup analysis of all patients. **(A)** Forest plot of overall survival and progression-free survival **(B)** in all patients. The point represents the HR of each subgroup, and the horizontal line represents the 95%CI of HR. The dash line represents the HR=1.
