# Supplementary figures and images for: Lenvatinib versus bevacizumab when combined with PD-1/L1 inhibitor and hepatic arterial infusion chemotherapy in unresectable hepatocellular carcinoma
Source: Front Immunol. 2025 May 23;16:1573098. doi: 10.3389/fimmu.2025.1573098 (PMC12141330; doi:10.3389/fimmu.2025.1573098)

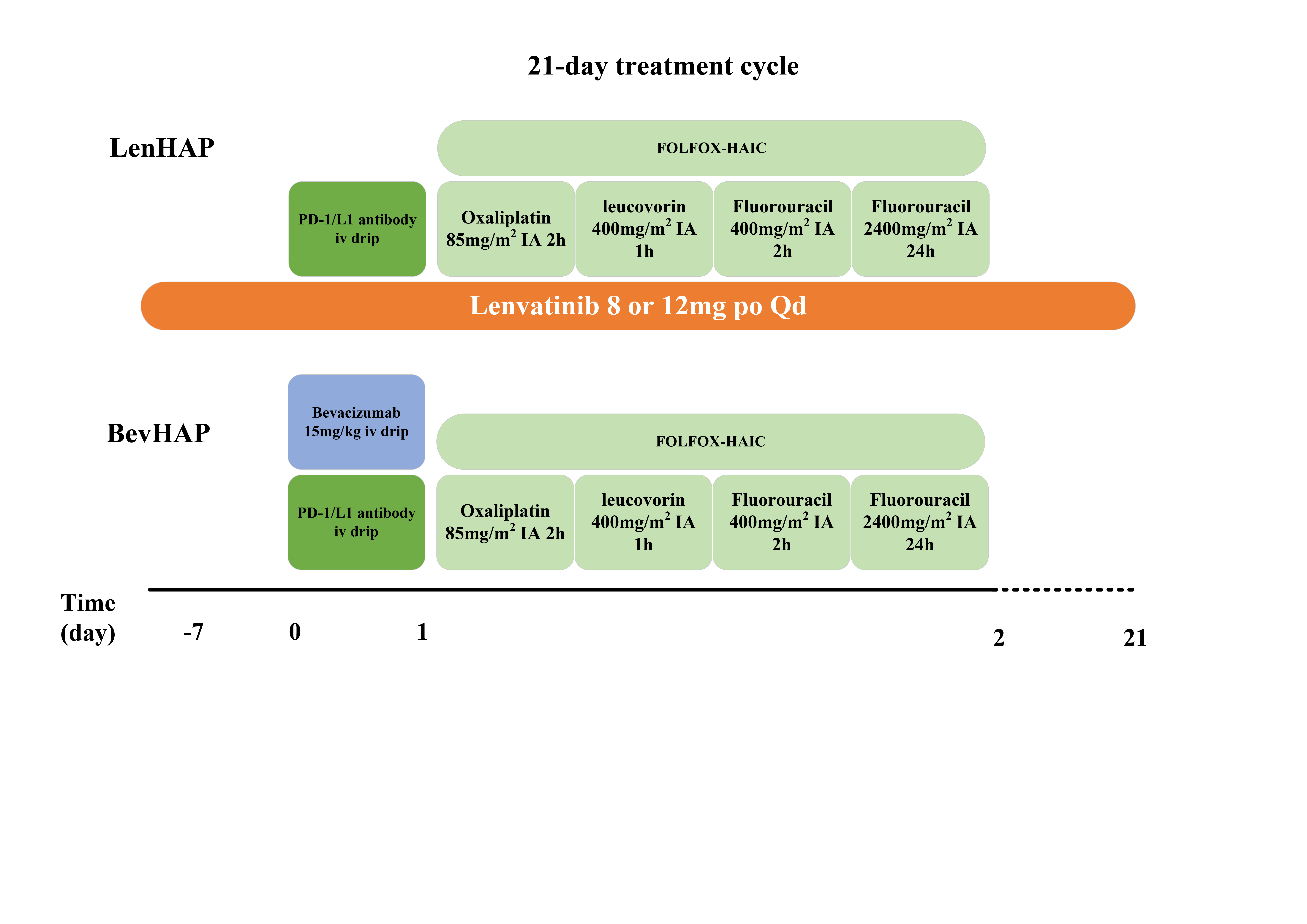

Supplement: Supplementary Figure 1 — Graphical abstract of two treatment groups. [file Image1.tif]

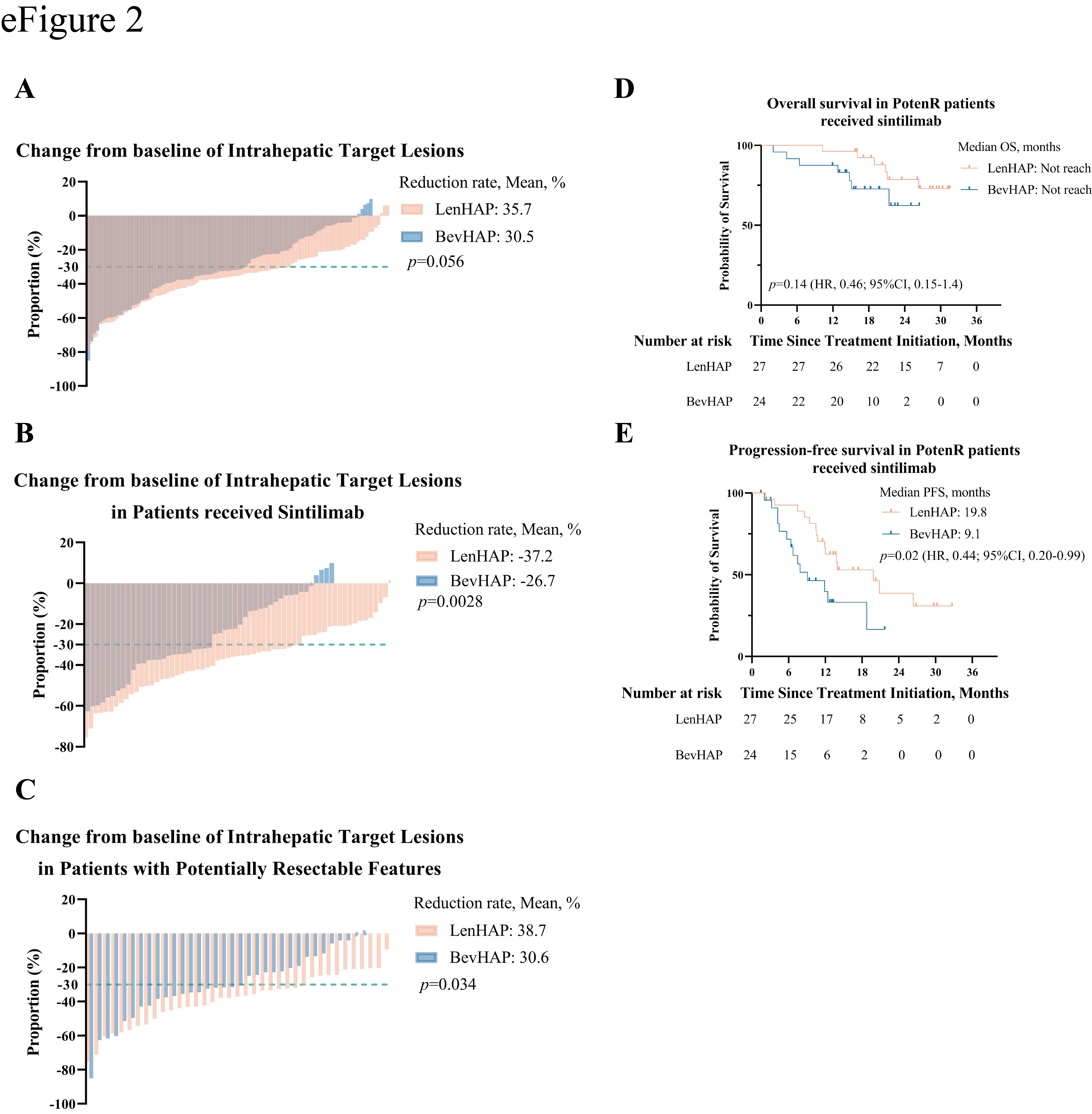

Supplement: Supplementary Figure 2 — Reduction rate of intrahepatic target lesions, and survival analysis in PotenR patients received sintilimab. (A) Reduction rate of intrahepatic target lesions per RECIST v1.1 in all patients who achieved disease control, in patients who received sintilimab and achieved disease control (B), and in PotenR patients who achieved disease control (C). (D) Kaplan-Meier curves of overall survival and progression-free survival (E) in PotenR patients who received sintilimab. [file Image2.tif]

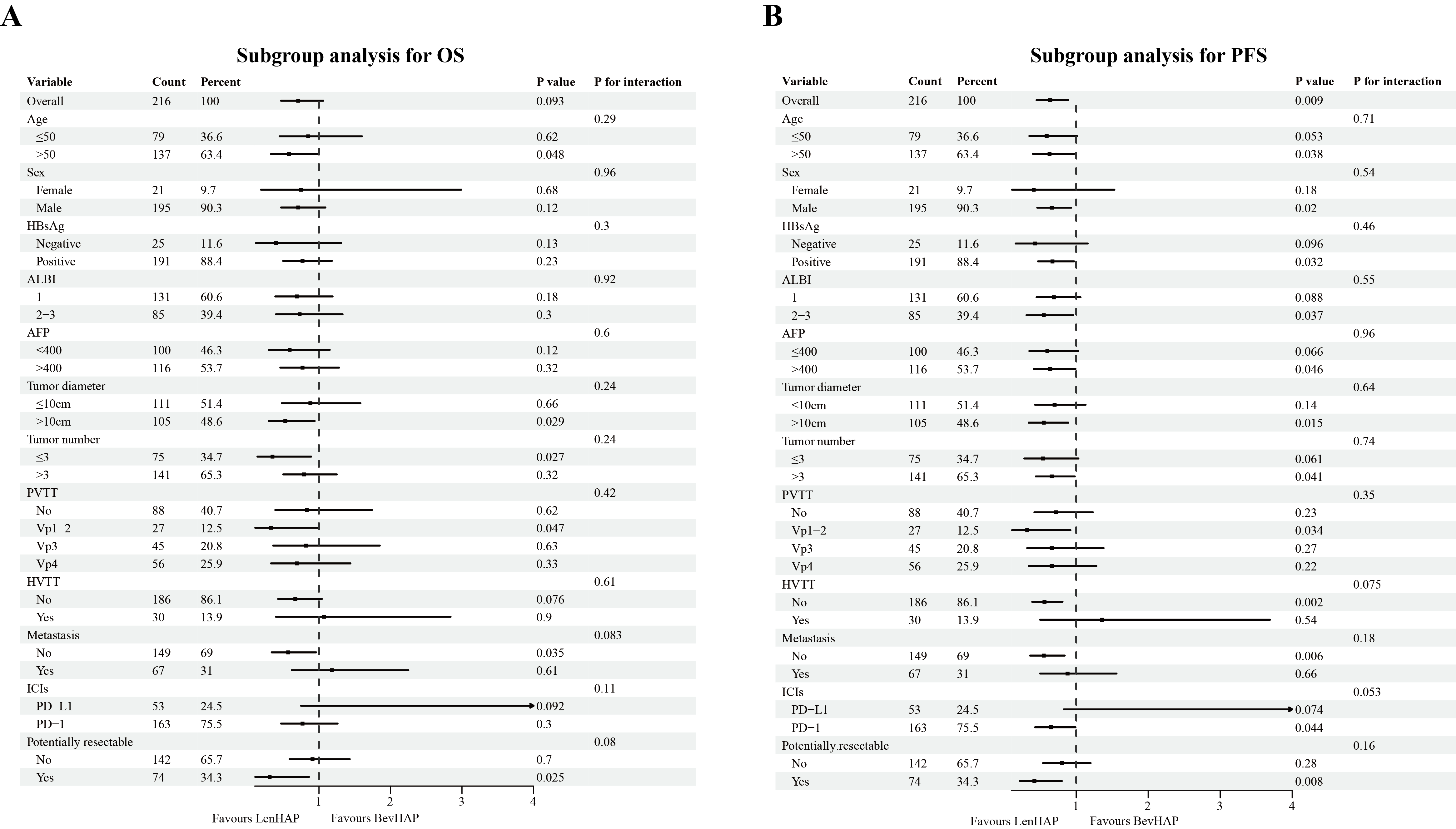

Supplement: Supplementary Figure 3 — Subgroup analysis of all patients. (A) Forest plot of overall survival and progression-free survival (B) in all patients. The point represents the HR of each subgroup, and the horizontal line represents the 95%CI of HR. The dash line represents the HR=1. [file Image3.tif]
