# Supplementary material for: Lenvatinib versus bevacizumab when combined with PD-1/L1 inhibitor and hepatic arterial infusion chemotherapy in unresectable hepatocellular carcinoma
Source: Front Immunol. 2025 May 23;16:1573098. doi: 10.3389/fimmu.2025.1573098 (PMC12141330; doi:10.3389/fimmu.2025.1573098)
Supplement: Supplementary file 4 [file DataSheet1.docx]

**eMethods**

**Definition of outcomes**

OS was defined as the time from the commencement of treatment to death from any cause or the date of the last follow-up if the patient was alive. PFS was the interval from the commencement of treatment to the first documented disease progression according to RECIST v1.1 or death from any cause, whichever occurred first. ORR was the percentage of patients whose best overall response was complete response (CR) or partial response (PR) that was maintained for at least 4 weeks from the first radiological confirmation, and DCR was the proportion of patients who had a CR, PR, or stable disease (SD). DoR was defined as the time from the first documented CR or PR to disease progression or death. Adverse events were evaluated by vital signs and clinical laboratory test results and assessment of the incidence and severity of adverse events according to the National Cancer Institute [NCI] Common Terminology Criteria for Adverse Events, version 5.0.

**Treatments**

Included patients received the treatment of lenvatinib (LenHAP) or bevacizumab (BevHAP) combined with ICIs (programmed death 1/ programmed death 1 ligand 1 antibody, PD-1/L1 antibody) and HAIC as the first-line treatment.

In the LenHAP group, patients initiated lenvatinib (12 mg/day [for bodyweight ≥60kg] or 8 mg/day [for bodyweight <60kg]) 3-7 days before initial HAIC to confirm tolerability and then underwent 21-day treatment cycles of lenvatinib (day 1 to day 21), PD-1/L1 antibody (day1), and HAIC (day 1 to day 21) with the FOLFOX regimen (oxaliplatin 85 mg/m^2^ from hour 0 to 2 on day 1; leucovorin 400 mg/m^2^ from hour 2 to 3 on day 1; 5-fluorouracil 400 mg/m^2^ bolus at hour 3; and 2400 mg/m^2^ over 24 hours on days 1 and 2). PD-1/L1 antibody was administered before each HAIC cycle.

In the BevHAP group, patients received 15 mg/kg body weight of bevacizumab and PD-1/L1 antibody intravenously followed by HAIC every 21 days.

The procedure of HAIC was shown as follows. An arterial catheter was inserted using an image-guided procedure through the femoral artery. The femoral artery was percutaneously punctured using the Seldinger’s technique. A 5 French catheter will be inserted into the celiac trunk or superior mesenteric artery for arteriography. Then, a 2.7 French microcatheter will be superselectively placed into the feeding arteries of the tumor and the tumor thrombus. If the tumors simultaneously accept blood supply from the celiac trunk and superior mesenteric artery, the microcatheter will be placed into the largest tumor feeding arteries. When blood flow into the gastroduodenal artery was confirmed by micro-catheter angiography, the route was embolized with a coil or micro-coil to prevent reflux of chemotherapeutic drugs to the stomach and duodenum. The peripheral end of the micro-catheter will be locked with a heparin lock (10 ml, 10,000 units, 1: 1,000 dilution) to prevent clotting of the catheter. The peripheral part of the catheter exposed outside the body will be covered with medical sterile gauze and fastened on the skin of the thigh using medical rubberized fabric and a bandage. Then, the patient will be transferred to the ward and confined to bed for 24 hours. After confirming the location of the tips of the microcatheter by bed side X-ray radiography, the microcatheter was marked in vitro and connected to the artery infusion pump to administer the chemotherapy agent. When the mark changed, bedside X-ray radiography was also conducted to confirm the location of the catheter tip. If dislocation of the catheter tip was confirmed, the patient was transferred to the digital subtraction angiography room to correct the location of the catheter tip. After HAIC is completed, the catheter and sheath will be removed. No implanted port catheter system was used and repetitive femoral artery puncture and catheterization was performed in the next HAIC cycle.

Within treatment, the following parameters were collected every one cycle: signs, symptoms, hematology parameters, blood biochemistry, urinalysis, coagulation, tumor markers (AFP, PIVKA-II, CA199), etc. Upper abdomen-enhanced CT (MRI is also acceptable) and chest-enhanced CT was performed every 2 cycles.

**Dose reduction, interruption, and discontinuation of therapy**

Dose reductions were permitted for lenvatinib (to 8 mg and 4 mg/day, or 4 mg every other day) in the event of toxicity.^1^ The decision to delay lenvatinib and PD-1/L1 antibodies follows local standards of care as guided by the locally approved product label. Dose reductions, treatment interruptions, and discontinuations of HAIC were performed according to our previous study.^2^ HAIC was delayed until recovery if neutrophil count less than 1200 cells/μL, platelet count less than 60,000 platelets/μL, a total bilirubin level exceeding 30 μmol/L, an albumin level less than 30 g/L, or serum creatinine up to 1.5 times the institutional upper limit of normal. The 5-fluorouracil dose was decreased to a 300 mg/m^2^ bolus and 1800 mg/m^2^/cycle continuous infusion if grade 3 or 4 diarrhea or stomatitis, skin toxicity, or other grade 3 major organ drug-related toxicity occurred. The oxaliplatin dose was decreased to 65 mg/m^2^/cycle if grade 3 or 4 neutropenia or thrombocytopenia, any other grade 3 major organ drug-related toxicity, or paresthesia associated with pain occurred.

The study treatment was stopped in cases of tumor progression as defined by RECIST version 1.1^3^, intolerable toxicity, study treatment delays of more than 30 days, the need for another anticancer treatment (such as surgery) at the physician’s discretion, or at the patient’s request. HAIC was also discontinued in cases of the disappearance of any arterial enhancement in all intrahepatic lesions or technical difficulty. Then, the patients entered the post-study treatment period.

**Post-study treatment**

The choice of post-study treatment was determined according to the tumor stage and the results of discussions by a local multidisciplinary team. For patients whose residual tumors could be safely removed by surgery or ablated by radiofrequency ablation, the corresponding treatment was recommended. If the tumors were completely devascularized, the patients were recommended to discontinue HAIC and receive lenvatinib plus PD-1/L1 antibodies. For other patients with tumor progression, systemic treatment (sorafenib, regorafenib, or atezolizumab plus bevacizumab) was recommended. Best supportive care was provided to patients with terminal hepatocellular carcinoma, Child-Pugh C liver function, or ECOG score >2.

**eResults**

**Results of subgroup analyses**

The results of subgroup analyses indicated that patients with PotenR, age over 50, tumor diameter over 10cm, tumor number less than 3, Vp1-2, and absent with metastasis significantly benefited OS from LenHAP. Male patients absent with HVTT and metastasis, as well as those with PotenR, age over 50, HBV infection, ALBI grade 2-3, serum AFP level over 400, tumor diameter over 10cm, tumor number over 3, Vp1-2 and PD-1 antibody therapy significantly benefited PFS from LenHAP.

**Patients treated with sintilimab (PD-1 antibody) (n=118)**

The median OS was similar between the two groups (26.4 vs 24.7 months, *p*=0.28) (Figure 4A). Compared with BevHAP group, the LenHAP group presented with significantly longer PFS (14.0 vs 9.0 months, *p*=0.029) (Figure 4B), and significantly higher ORR per RECIST v1.1 (65.1% vs 45.5%, *p*=0.032) or mRECIST (90.5% vs 67.3, *p*=0.002) (eTable 3). In patients with DCR, the mean reduction rate per RECIST v1.1 for intrahepatic targeted lesions was 37.2% and 26.7% in the respective groups (*p*=0.0028) (eFigure 2B). The conversion resection rate was 27.0% in LenHAP group and 12.7% in BevHAP group (*p*=0.055). The tumor number, serum AFP level and PotenR were the independent risk factors for OS, while the treatment group and serum AFP level were the independent risk factors for PFS (eTable 4).

Reference

1. Kudo M, Finn RS, Qin S, et al: Lenvatinib versus sorafenib in first-line treatment of patients with unresectable hepatocellular carcinoma: a randomised phase 3 non-inferiority trial. Lancet 391:1163-1173, 2018

2. He M, Li Q, Zou R, et al: Sorafenib Plus Hepatic Arterial Infusion of Oxaliplatin, Fluorouracil, and Leucovorin vs Sorafenib Alone for Hepatocellular Carcinoma With Portal Vein Invasion: A Randomized Clinical Trial. JAMA Oncol, 2019

3. Eisenhauer EA, Therasse P, Bogaerts J, et al: New response evaluation criteria in solid tumours: revised RECIST guideline (version 1.1). Eur J Cancer 45:228-47, 2009
