# Supplementary material for: Lenvatinib versus bevacizumab when combined with PD-1/L1 inhibitor and hepatic arterial infusion chemotherapy in unresectable hepatocellular carcinoma
Source: Front Immunol. 2025 May 23;16:1573098. doi: 10.3389/fimmu.2025.1573098 (PMC12141330; doi:10.3389/fimmu.2025.1573098)
Supplement: Supplementary file 5 [file Table1.docx]

eTable 1. Treatment administration

|  | LenHAP (n=108) | BevHAP (n=108) | *p* |
| --- | --- | --- | --- |
| Treatment cycles |  |  | 0.55 |
| Mean (SD) | 3.0 (1.1) | 3.2 (1.5) |  |
| Median (IQR) | 3 (2-4) | 3 (2-4) |  |
| Subsequent treatment |  |  |  |
| Resection | 21 | 10 | 0.033 |
| Ablation | 7 | 4 | 0.35 |
| TACE | 19 | 32 | 0.037 |
| Systemic chemotherapy | 19 | 8 | 0.024 |
| Radiotherapy | 4 | 3 | 1.0 |
| Anti-angiogenic drugs changed | 15 | 9 | 0.19 |
| Regorafenib | 7 | 1 | 0.065 |
| Rivoceranib | 7 | 0 | 0.014 |
| Lenvatinib | 1 | 8 | 0.035 |
| Bevacizumab | 2 | 0 | 0.50 |
| PD-1/PD-L1 changed | 10 | 4 | 0.097 |
| Tislelizumab | 1 | 1 | 1.0 |
| Camrelizumab | 8 | 0 | 0.007 |
| Atezolizumab | 1 | 0 | 1.0 |
| Cadonilimab | 1 | 2 | 1.0 |
| Sintilimab | 0 | 1 | 1.0 |

eTable 2. Univariate and multivariate analysis of OS and PFS

|  | Overall survival | | | Progression-free survival | | |
| --- | --- | --- | --- | --- | --- | --- |
|  | Univariate analysis | Multivariate analysis | | Univariate analysis | Multivariate analysis | |
|  | P1 | HR (95%CI) | P2 | P1 | HR (95%CI) | P2 |
| Group (LenHAP vs BevHAP) | 0.093 | - | - | 9.1*10^-3^ | 0.60 (0.42-0.85) | 4.1*10^-3^ |
| Age (>50 vs ≤50) | 0.055 | - | - | 2.8*10^-3^ | 0.62 (0.43-0.89) | 9.5*10^-3^ |
| Sex (Male vs Female) | 0.43 | - | - | 0.66 | - | - |
| HbsAg (Positive vs Negative) | 0.64 | - | - | 0.95 | - | - |
| Tumor size (>10cm vs ≤10cm) | 0.32 | - | - | 0.73 | - | - |
| Tumor number (>3 vs ≤3) | 2.5*10^-4^ | 1.8 (1.1-3.0) | 0.019 | 1.9*10^-3^ | 1.7 (1.2-2.5) | 7.7*10^-3^ |
| PVTT (presence vs absence) | 4.0*10^-3^ | 1.3 (0.83-2.2) | 0.23 | 0.48 | - | - |
| HVTT (presence vs absence) | 0.072 | - | - | 0.78 | - | - |
| Metastasis (presence vs absence) | 8.1*10^-4^ | 1.6 (1.1-2.6) | 0.030 | 1.2*10^-4^ | 1.6 (1.1-2.4) | 0.014 |
| AFP (>400 vs ≤400) | 6.5*10^-3^ | 1.4 (0.90-2.1) | 0.14 | 1.6*10^-3^ | 1.5 (1.1-2.2) | 0.023 |
| ALBI (Grade 2-3 vs Grade 1) | 0.02 | 1.4 (0.94-2.1) | 0.10 | 0.8 | - | - |
| Potential resectable (Yes vs No) | 4.8*10^-6^ | 0.5 (0.28-0.91) | 0.022 | 2.4*10^-3^ | 0.88 (0.59-1.3) | 0.55 |

eTable 3. Baseline characteristics for patients received sintilimab

|  | LenHAP (n=63) | BevHAP (n=55) | *p* |
| --- | --- | --- | --- |
| Age, year, mean (SD) |  |  | 0.54 |
| ≤50 | 24 (38.1%) | 18 (32.7%) |  |
| ＞50 | 39 (61.9%) | 37 (67.3%) |  |
| Sex |  |  | 0.58 |
| male | 58 (92.1%) | 49 (89.1%) |  |
| female | 5 (7.9%) | 6 (10.9%) |  |
| HBsAg |  |  | 0.27 |
| Positive | 53 (84.1%) | 50 (90.9%) |  |
| Negative | 10 (15.9%) | 5 (9.1%) |  |
| Child-Pugh grade |  |  | 0.18 |
| A | 62 (98.4%) | 51 (92.7%) |  |
| B | 1 (1.6%) | 4 (7.3%) |  |
| Tumor size, cm, mean (SD) | 11.1 (3.9) | 9.1 (3.9) | 0.036 |
| ≤10 | 16 (42.9%) | 32 (58.2%) | 0.097 |
| ＞10 | 36 (57.1%) | 23 (41.8%) |  |
| Tumor number |  |  | 0.83 |
| ≤3 | 24 (38.1%) | 22 (40.0%) |  |
| ＞3 | 39 (61.9%) | 33 (60.0%) |  |
| PVTT |  |  | 0.51 |
| Vp0  Vp1-2  Vp3  Vp4 | 30 (47.6%)  4 (6.3%)  14 (22.2%)  15 (23.8%) | 28 (50.9%)  7 (12.7%)  8 (14.5%)  12 (21.8%) |  |
| HVTT |  |  | 1.0 |
| No | 56 (88.9%) | 48 (87.3%) |  |
| Yes | 7 (11.1%) | 7 (12.7%) |  |
| Metastasis |  |  | 0.14 |
| No | 50 (79.4%) | 37 (67.3%) |  |
| Yes | 13 (20.6%) | 18 (32.7%) |  |
| AFP, ng/ml, median (IQR) | 88.2 (8.3-1207) | 550 (9.4-7387) | 0.37 |
| ≤400 | 28 (44.4%) | 27 (49.1%) | 0.61 |
| ＞400 | 35 (55.6%) | 28 (50.9%) |  |
| Potentially resectable  No  Yes | 36 (57.1%)  27 (42.9%) | 31 (56.4%)  24 (43.6%) | 0.93 |
| ORR (RECIST v1.1) |  |  | 0.032 |
| Yes | 41 (65.1%) | 25 (45.5%) |  |
| No | 22 (34.9%) | 30 (54.5%) |  |
| ORR (mRECIST) |  |  | 0.002 |
| Yes | 57 (90.5%) | 37 (67.3%) |  |
| No | 6 (9.5%) | 18 (32.7%) |  |
| Intrahepatic ORR (RECIST v1.1) |  |  | 0.012 |
| Yes | 43 (68.3%) | 25 (45.5%) |  |
| No | 20 (31.7%) | 30 (54.5%) |  |
| Intrahepatic ORR (mRECIST) |  |  | <0.001 |
| Yes | 59 (93.7%) | 37 (67.3%) |  |
| No | 4 (6.3%) | 18 (32.7%) |  |

eTable 4. Univariate and multivariate analysis of OS and PFS in patients received sintilimab

|  | Overall survival | | | Progression-free survival | | |
| --- | --- | --- | --- | --- | --- | --- |
|  | Univariate analysis | Multivariate analysis | | Univariate analysis | Multivariate analysis | |
|  | P1 | HR (95%CI) | P2 | P1 | HR (95%CI) | P2 |
| Group (LenHAP vs BevHAP) | 0.28 | - | - | 0.031 | 0.53 (0.33-0.85) | 9.0*10^-3^ |
| Age (>50 vs ≤50) | 0.075 | - | - | 7.5*10^-3^ | 0.56 (0.34-0.92) | 0.23 |
| Sex (Male vs Female) | 0.46 | - | - | 0.40 | - | - |
| HbsAg (Positive vs Negative) | 0.51 | - | - | 0.38 | - | - |
| Tumor size (>10cm vs ≤10cm) | 0.18 | - | - | 0.22 | - | - |
| Tumor number (>3 vs ≤3) | 1.3*10^-3^ | 2.2 (1.2-4.4) | 0.017 | 6.1*10^-3^ | 1.9 (1.2-3.2) | 0.10 |
| PVTT (presence vs absence) | 6.6*10^-3^ | 1.4 (0.72-2.5) | 0.35 | 0.38 | - | - |
| HVTT (presence vs absence) | 0.72 | - | - | 0.13 | - | - |
| Metastasis (presence vs absence) | 0.072 | - | - | 0.37 | - | - |
| AFP (>400 vs ≤400) | 5.1*10^-3^ | 1.9 (1.1-3.5) | 0.033 | 3.0*10^-3^ | 1.9 (1.2-3.1) | 0.011 |
| ALBI (Grade 2-3 vs Grade 1) | 0.49 | - | - | 0.55 | - | - |
| Potential resectable (Yes vs No) | 1.7*10^-4^ | 0.5 (0.22-0.90) | 0.024 | 0.088 | - | - |

eTable 5. Baseline characteristics for PotenR patients

|  | LenHAP (n=38) | BevHAP (n=36) | *p* |
| --- | --- | --- | --- |
| Age, year, mean (SD) |  |  | 0.89 |
| ≤50 | 10 (26.3%) | 10 (27.8%) |  |
| ＞50 | 28 (73.7%) | 26 (72.2%) |  |
| Sex |  |  | 1.0 |
| male | 35 (92.1%) | 34 (94.4%) |  |
| female | 3 (7.9%) | 2 (5.6%) |  |
| HBsAg |  |  | 0.35 |
| Positive | 31 (81.6%) | 33 (91.7%) |  |
| Negative | 7 (18.4%) | 3 (8.3%) |  |
| Child-Pugh grade |  |  | 1.0 |
| A | 37 (97.4%) | 36 (100%) |  |
| B | 1 (2.6%) | 0 (0%) |  |
| Tumor size, cm, mean (SD) | 11.1 (3.9) | 9.1 (3.9) | 0.036 |
| ≤10 | 16 (42.1%) | 24 (66.7%) | 0.034 |
| ＞10 | 22 (57.9%) | 12 (33.3%) |  |
| Tumor number |  |  | 0.36 |
| ≤3 | 22 (57.9%) | 17 (47.2%) |  |
| ＞3 | 16 (42.1%) | 19 (52.8%) |  |
| PVTT |  |  | 0.66 |
| Vp0  Vp1-2  Vp3  Vp4 | 25 (65.8%)  7 (18.4%)  5 (13.2%)  1 (2.6%) | 25 (69.4%)  4 (11.1%)  7 (19.4%)  0 (0%) |  |
| HVTT |  |  | 1.0 |
| No | 35 (92.1%) | 33 (91.7%) |  |
| Yes | 3 (7.9%) | 3 (8.3%) |  |
| Lymph node metastasis |  |  | 0.68 |
| No | 34 (89.5%) | 34 (94.4%) |  |
| Yes | 4 (10.5%) | 2 (5.6%) |  |
| AFP, ng/ml, median (IQR) | 88.2 (8.3-1207) | 550 (9.4-7387) | 0.37 |
| ≤400 | 26 (68.4%) | 15 (44.4%) | 0.037 |
| ＞400 | 12 (31.6%) | 20 (55.6%) |  |
| Intrahepatic ORR (RECIST v1.1) |  |  | 0.11 |
| Yes | 27 (71.1%) | 19 (52.8%) |  |
| No | 11 (28.9%) | 17 (47.2%) |  |
| Intrahepatic ORR (mRECIST) |  |  | 0.053 |
| Yes | 37 (97.4%) | 30 (83.3%) |  |
| No | 1 (2.6%) | 6 (16.7%) |  |

eTable 6. Univariate and multivariate analysis of OS and PFS in PotenR patients

|  | Overall survival | | | Progression-free survival | | |
| --- | --- | --- | --- | --- | --- | --- |
|  | Univariate analysis | Multivariate analysis | | Univariate analysis | Multivariate analysis | |
|  | P1 | HR (95%CI) | P2 | P1 | HR (95%CI) | P2 |
| Group (LenHAP vs BevHAP) | 0.025 | 0.37 (0.14-1.0) | 0.057 | 8.3*10^-3^ | 0.46 (0.24-0.88) | 0.018 |
| Age (>50 vs ≤50) | 0.28 | - | - | 3.5*10^-3^ | 0.38 (0.20-0.742) | 4.2*10^-3^ |
| Sex (Male vs Female) | 0.81 | - | - | 0.46 | - | - |
| HbsAg (Positive vs Negative) | 1.0 | - | - | 0.93 | - | - |
| Tumor size (>10cm vs ≤10cm) | 0.41 | - | - | 0.82 | - | - |
| Tumor number (>3 vs ≤3) | 0.02 | 3 (1.0-8.4) | 0.042 | 0.15 | - | - |
| PVTT (presence vs absence) | 0.39 | - | - | 0.76 | - | - |
| HVTT (presence vs absence) | 0.83 | - | - | 0.36 | - | - |
| Metastasis (presence vs absence) | 0.40 | - | - | 0.95 | - | - |
| AFP (>400 vs ≤400) | 0.33 | - | - | 8.3*10^-3^ | 2.0 (1.1-3.7) | 0.031 |
| ALBI (Grade 2-3 vs Grade 1) | 0.18 | - | - | 0.13 | - | - |

eTable 7. Baseline characteristics for potentially unresectable patients with surgical resection.

| ID | Age | Sex | BCLC stage | Before Treatment | | | | | After treatment | | OS, months | PFS, months |
| --- | --- | --- | --- | --- | --- | --- | --- | --- | --- | --- | --- | --- |
|  |  |  |  | Tumor diameter, cm | Tumor number | Unilobar Confinement | Vascular invasion | Metastasis | Tumor diameter, cm | Vascular invasion |  |  |
| 1 | 41 | Male | C | 7.46 | 4 | Yes | PVTT Vp4 | No | 2.73 | PVTT Vp3 | 24.8 | 15.0 |
| 2 | 41 | Male | C | 13.00 | 1 | Yes | PVTT Vp4 | No | 9.70 | PVTT Vp3 | 14.5 | 8.1 |
